# Human Breast Cancer Cell Lines Co-Express Neuronal, Epithelial, and Melanocytic Differentiation Markers *In Vitro* and *In Vivo*

**DOI:** 10.1371/journal.pone.0009712

**Published:** 2010-03-16

**Authors:** Qingbei Zhang, Hanli Fan, Jikun Shen, Robert M. Hoffman, H. Rosie Xing

**Affiliations:** 1 Department of Pathology, Cellular and Radiation Oncology, The University of Chicago, Chicago, Illinois, United States of America; 2 Ludwig Center for Metastasis Research, The University of Chicago, Chicago, Illinois, United States of America; 3 AntiCancer, Inc., San Diego, California, United States of America; 4 Department of Surgery, University of California San Diego, San Diego, California, United States of America; Health Canada, Canada

## Abstract

Differentiation programs are aberrant in cancer cells allowing them to express differentiation markers in addition to their tissue of origin. In the present study, we demonstrate the multi-lineage differentiation potential of breast cancer cell lines to express multiple neuronal/glial lineage-specific markers as well as mammary epithelial and melanocytic-specific markers. Multilineage expression was detected in luminal (MCF-7 and SKBR3) and basal (MDA-MB-231) types of human breast cancer cell lines. We also observed comparable co-expression of these three cell lineage markers in MDA-MB-435 cells *in vitro*, in MDA-MB-435 primary tumors derived from parental and single cell clones and in lung metastases *in vivo*. Furthermore, ectoderm multi-lineage transdifferentiation was also found in human melanoma (Ul-MeL) and glioblastoma cell lines (U87 and D54). These observations indicate that aberrant multi-lineage transdifferentiation or lineage infidelity may be a wide spread phenomenon in cancer.

## Introduction

Cancer diagnosis of the tissue origin of metastatic lesions, especially those from occult primary tumors, relies heavily on the expression of cellular or tissue differentiation markers. Emerging clinical and pre-clinical evidence show that the differentiation programs are aberrant in cancer cells allowing them to express differentiation markers beyond their tissue of origin [1]–[3]. These observations have implications for both cancer research and clinical management of cancer. However, this property has not been thoroughly examined in human cancer cell lines that are frequently used in cancer research.

A microarray analysis has indicated that the gene expression pattern of the human MDA-MB-435 [4] resembles that of human melanoma cell lines [5]. Since then, additional evidence has shown the ability of MDA-MB-435 cells to express melanocytic markers [6]–[8]. However, upon induction with heregulin *in vitro*, MDA-MB-435 cells undergo sufficient mammary epithelial differentiation to produce milk lipid droplets and to express β-casein mRNA [9]. Additionally, enhanced expression of NM23-H1 metastasis suppressor gene leads to the formation of organized mammary acinus-like sphere in 3D culture and the expression of sialomucin (epithelial membrane antigen, EMA) [10]. A recent investigation confirmed the ability of MDA-MB-435 cells to co-express markers of mammary epithelium and melanocytes both *in vitro* and *in vivo*, and postulated committing lineage infidelity as an underlying mechanism for the observed dual lineage transdifferentiation [11]. Furthermore, another recent study reported the robust expression of melanocyte-related genes in a variety of breast cancer cell lines including MDA-MB-435, and more importantly in freshly resected and histopathologically confirmed human breast cancer specimens [12].

Here, we report the expression of multiple neuronal/glial lineage-specific markers by MDA-MB-435 cultured cells *in vitro* and in primary tumors and lung metastases *in vivo* in addition to the reported expression of epithelial and melanocytic markers. Furthermore, we observed the co-expression of three ectoderm cell lineage markers in other luminal (MCF-7 and SKBR3) and basal (MDA-MB-231) types of human breast cancer cell lines, as well as in human melanoma and glioblastoma cancer cell lines generated from tumors of ectoderm origin.

These observations indicate that while terminal differentiation to the anticipated cellular type is compromised in the cancerous state, aberrant multi-lineage transdifferentiation or lineage infidelity may be a wide spread in cancer phenomenon.

## Materials and Methods

### Cell culture

The MDA-MB-435s, MDA-MB-231, MCF-7 and MCF-10A cell lines were obtained from ATCC (Manassa, VA). SKBR3 cells were gifts from Dr. Suzanne Conzen, University of Chicago. Ul-Mel, U87, and D54 were from gifts from Dr. Ralph Weichselbaum, University of Chicago. All cells were cultured in DMEM high glucose (Hyclon, Logan, Utah) supplemented with 10% FBS and 1% penicillin/streptomycin. MDA-MB-435-GFP derivative cell lines were generated from single cell cloning through initial seeding of single cells in 96-well plates and subsequent expansion of cell numbers. MDA-MB-435-GFP-L cell lines were generated from resected lungs that harbor pleural metastases derived from tail-vein injected MDA-MB-435-GFP cells and purified using G418 for the selection of GFP expression.

### PCR analysis of lineage markers expression

Total RNA was isolated using TRIZOL (Applied Biosystems Inc. Foster City, CA) according to the manufacturer's instructions, followed by DNAse treatment (Promega, Madison, WI). cDNA was reverse transcribed from 2 µg of total RNA using random primer method (Applied Biosystems Inc.) according to the manufacturer's instructions. PCR amplification was performed by incubation at 94°C for 1 min, followed by 32 cycles of 94°C for 30 s, 55°C for 30s, and 72°C for 25s. Amplified products were separated and visualized on a 0.8% agarose gel. TBP was used as a loading control.

The sequence for the primer sets used are as follows: Nestin (F: aacagcgacggaggtctcta; R: ttctcttgtcccgcagactt), EMA (F: tcccagcaccgactactacc; R: cagctgcccgtagttctttc), ESA (F: ggaagctgagtgcaagaagg; R: gctgcacaacctcaatctca), TRY (F: tacggcgtaatcctggaaac; R: attgtgcatgctgctttgag), Melan-A (F: gctcatcggctgttggtatt; R: ataagcaggtggagcattgg), MITF (F: aactcatgcgtgagcagatg; R: tacttggtggggttttcgag), GFAP (F: acatcgagatcgccacctac; R: atctccacggtcttcaccac), TUBB3 (F: cagatgttcgatgccaagaa; R: gggatccactccacgaagta), CK19 (F: tttgagacggaacaggctct; R: aatccacctccacactgacc), CK17 (F: gctgctacagctttggctct; R: tcacctccagctcagtgttg), and TBP (F: tataatcccaagcggtttgc; R: cacagctccccaccatattc).

### Immunofluorescence analysis of differentiation marker expression in cultured cells

Five thousand unlabeled cells were plated in each chamber of 8-well chamber slides (BD) and grown for 48 hours. Cells were fixed with buffered formalin, permeabilized with 0.3% Triton X-100/PBS, blocked with 10% goat serum (Vector S-1000)/PBS and incubated with primary antibodies diluted in 2%BSA/1× PBS for 1 hour: ESA (Anaspec, San Jose, CA) (1∶100), melan-A (DAKO, Carpinteria, CA) (1∶100), Nestin (Abcam, Cambridge, MA) (1∶250) and GFAP (R &D, Minneapolis, MN) (1∶100). Thereafter, cells were incubated with Alexa 488-conjugated secondary antibodies (Molecular Probes) for 30 min, followed by mounting with Prolong Gold antifade reagent containing DAPI for nuclear counter-staining. Slides were photographed with a Leica SP-2 confocal microscope and images were processed with image J.

### Immunohistochemistry (IHC) analysis of differentiation marker expression in cultured cells, primary tumors, and lung metastases

All animal studies were carried out according to protocols approved by the IACUC Committee at the University of Chicago. Six-week-old female athymic Ncr/*nu/nu* mice (NCI-Frederick), 18 to 20 g, were used. For orthotopic tumor implantation, 5×10^6^ cultured MDA-MB-435 cells suspended in 0.1 ml of PBS were injected into the left inguinal mammary fat pad (m.f.p.). Tumors were harvested upon reaching ∼200 to 250 mm^3^, fixed in formalin and embedded in paraffin. To produce experimental lung metastases, 1×10^6^ cultured MDA-MB-435-GFP cells, suspended in 0.1 ml of PBS, were injected into the mouse tail vein. Twelve weeks later, mouse lungs were dissected and fixed in formalin and embedded in paraffin. The tumor and lung blocks were cut into 5-µm paraffin sections. For IHC analysis, tissue sections were deparafinized, rehydrated followed by antigen retrieval and treated with 3% hydrogen peroxide to block endogenous peroxidase activity. 1∶100 dilution of cytokeratin (DAKO, M3515), 1∶100 dilution of melan-A (DAKO M7196), 1∶100 GFAP (R & D System), and 1∶500 dilution of Nestin (Abcam, ab5968) were applied to the tissue slides and incubated for one hour. Envision+anti-mouse system was used for antigen-antibody detection. The slides were counter-stained with hematoxylin, air dried, and examined under light microscopy.

## Results and Discussion

### MDA-MB-435 cell lines derived from lung metastases exhibit morphology of neuronal/glial differentiation

**Figure 1:**
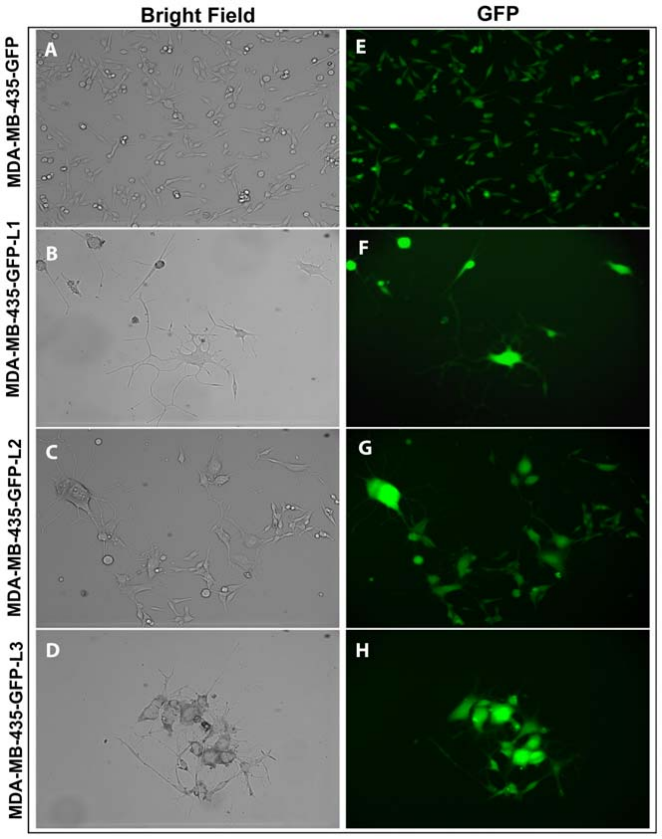
MDA-MB-435 lung cell lines derived from lung metastases display neuronal/glial differentiation morphology. **A** and **E**, parental MDA-MB-435-GFP cells. **B–D** and **F–H**, three representative MDA-MB-435-GFP-L cell lines exhibit morphological features of well-differentiated neuron/glial cells.

We generated an MDA-MB-435 cell line stably expressing green fluorescent protein (GFP) [13] and have been using this model routinely to produce experimental lung metastases for conducting *in vivo* imaging experiments (**data not shown**). We subsequently established MDA-MB-435-GFP sub-lines (MDA-MB-435-GFP-L) from pleural metastases harvested 12 weeks after tail vein injection of MDA-MB-435-GFP cells. In contrast to the spindle-shaped parental MDA-MB-435-GFP cells (**Figure 1A** and **E**), three representative MDA-MB-435-GFP-L cell lines displayed morphological features of differentiated neuron/glial cells such as the presence of polygonal soma (cell body), extended dendrites and axons, and cell-cell communication at synapses (**Figure 1 B–D** and **F–H**). Similar changes were also seen in non-GFP-labeled parental MDA-MB-435 cells freshly obtained from ATCC and in MDA-MB-435-GFP cell cultures set up at low seeding density (**data not shown**). Exhibition of diverse neuronal morphological features by MDA-MB-435 and MDA-MB-435-GFP-L cells lead us to hypothesize that in addition to undergoing aberrant melanocytic transdifferentiation, MDA-MB-435 cells are also capable of undergoing neuronal/glial-lineage transdifferentiation.

### mRNA transcripts of neuronal/glial, epithelial and melanocytic differentiation markers are co-expressed by MDA-MB-435 and other breast, melanoma and glioblastoma cancer cell lines

**Figure 2:**
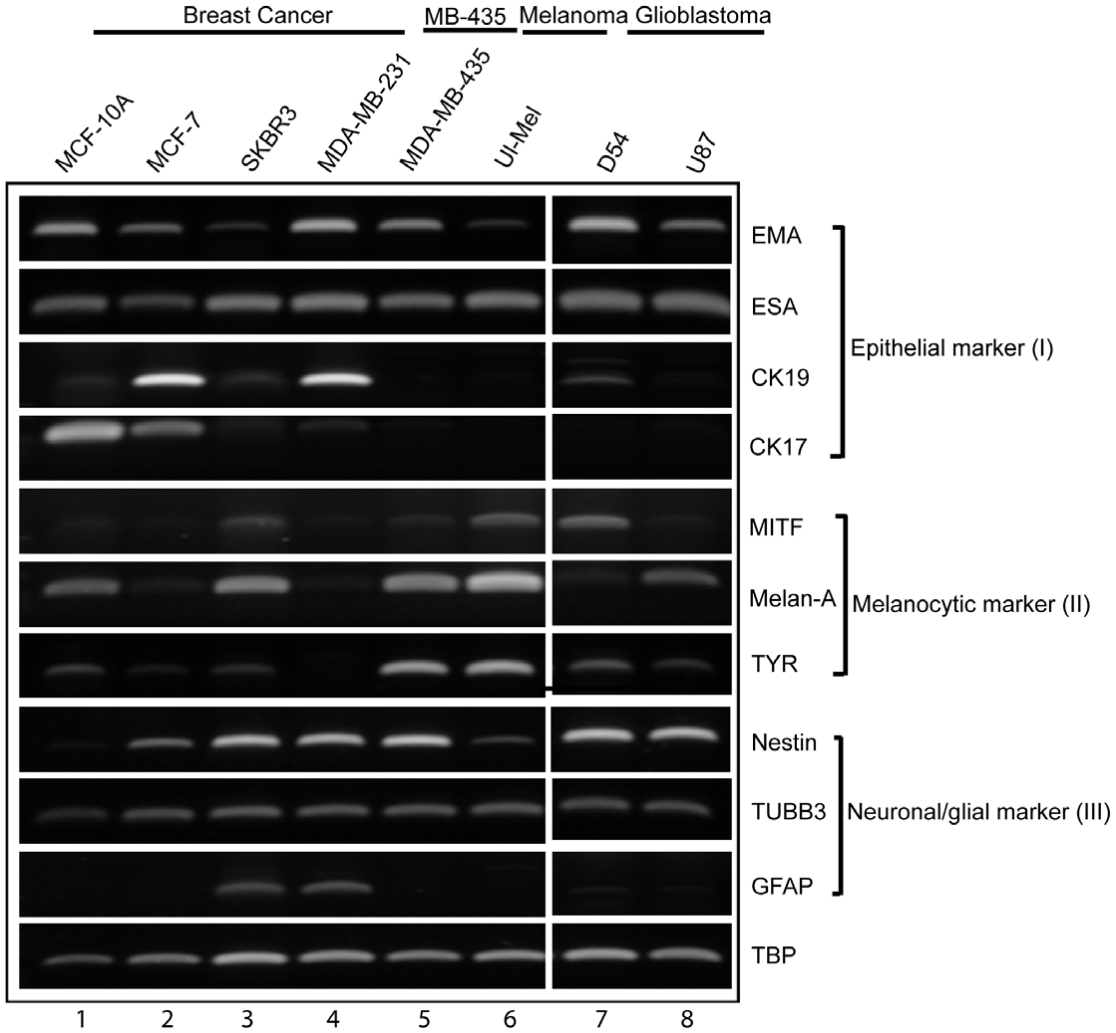
PCR analysis of mRNA expression of neuronal/glial, epithelial and melanocytic differentiation markers *in vitro*. Expression of epithelial (I), melanocytic (II) and neuronal/glial markers (III) by MCF-10A normal mammary epithelial cells (lane 1), breast cancer cell lines (lane 2–5), melanoma (lane 6), and glioblastoma cells (lane 7 and 8). TBP is used as a loading control.

Subsequently, we determined the expression of epithelial (ESA) and mammary epithelial (EMA, CK19, CK17) differentiation markers by RT-PCR analysis. In addition to the ER^−^/PR^−^/HER2^−^ MDA-MB-435 cells, we also included the ER^+^/PR^+^ luminal MCF-7 human breast cancer cell line (**Figure 2** **, lane 2**), the ER^−^/PR^−^/HER2^+^ luminal SKBR3 human breast cancer line (**lane 3**), and the highly metastatic basal ER^−^/PR^−^/HER2^−^ MDA-MB-231 (**lane 4**) breast cancer cell line as representatives of the major clinical subtypes of human breast cancer [14]. The immortalized MCF-10A cell line derived from normal basal mammary epithelium was chosen as a non-malignant control for comparison (**Figure 2** **, lane 1**). As expected, both the normal and malignant mammary epithelial cell lines expressed the epithelial markers EMA (epithelial membrane antigen, also known as MUC-1) and ESA (epithelial specific antigen) [**Figure 2(I)** **, lanes 1–5**]. While MCF-10A cells abundantly express the basal epithelial marker cytokeratin 17 (CK17) [**Figure 2(I)** **, lane 1**], CK17 was weakly expressed by basal metastatic MDA-MB-231 cancer cells [**Figure 2(I)** **, lane 4**], and was largely absent in MDA-MB-435 cells [**Figure 2(I)** **, lane 5**]. However, the basal marker CK17 was detected in luminal MCF-7 cells [**Figure 2(I)** **, lane 2**]. In comparison to CK17 expression, the luminal epithelial differentiation marker cytokeratin 19 (CK19) was expressed at high levels by luminal MCF-7 and weakly by SKBR3 cells [**Figure 2(I)** **, lanes 2–3**]. CK19 was also expressed by MDA-MB-231 cells [**Figure 2(I)** **, lane 4**] but was absent in MCF-10A non-malignant cells and in MDA-MB-435 cancer cells [**Figure 2(I)** **, lanes 1 and 5**]. The lack of expression of both CK17 and CK19 epithelial differentiation markers in cultured and non-induced MDA-MB-435 cells is consistent with its characterization as a poorly differentiated cancer cell line [4]. These results demonstrate that the expression of luminal and basal epithelial differentiation is aberrant in human breast cancer cell lines.

Thereafter, we determined the expression of three melanocytic differentiation markers (MITF, melan-A and tyrosinase) in breast cancer cell lines. We included human melanoma cell line Ul-MeL as a positive control for this analysis (**Figure 2** **, lanes 6**). Consistent with previous reports [3], [6]–[8], all three melanocytic markers were expressed by MDA-MB-435 cells [**Figure 2(II)** **, lane 5**]. The expression of all or a sub-set of the three markers was also detected in MCF-7, SKBR3 and normal MCF-10A cells [**Figure 2(II)** **, lanes 1–3**]. Weak but detectable expression of MITF and melan-A was seen in MDA-MB-231 cells, and tyrosinase (TYR) was nearly absent in MDA-MD-231 cells [**Figure 2(II)** **, lane 4**]. As anticipated, Ul-MeL melanoma cells express all three melanocytic markers [**Figure 2(II)** **, lanes 6**].

**Figure 3:**
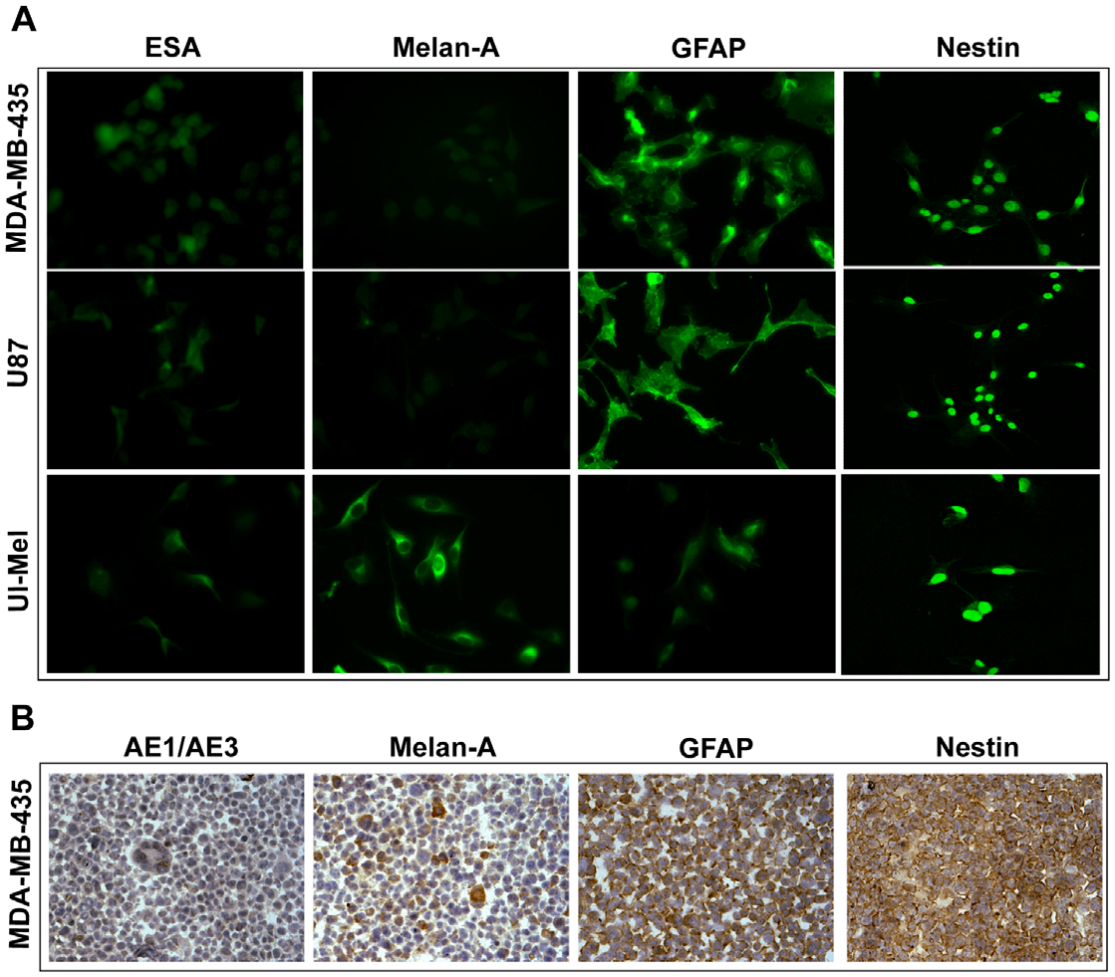
Determination of protein expression of lineage markers in cultured MDA-MB-435 cells. **A**, immunofluorescence staining of epithelial (ESA), melanocytic (melan-A) and neuronal/glial markers (GFAP and nestin) in cultured MDA-MB-435, U87 glioblastoma and Ul-Mel melanoma cancer cell lines. **B**, immunohistochemistry staining of lineage markers in embedded MDA-MB-435 cells. AE1/AE3: pan-cytokeratin epithelial marker.

Consistent with observed morphology of neuronal/glial cell differentiation **(** **Figure 1A, B** **)**, two neuronal markers, nestin and β3-tubulin, were robustly expressed by all four-breast cancer cell lines including MDA-MB-435 cells [**Figure 2(III)** **, lanes 2–5**]. In contrast, their mRNA expression was much lower in non-malignant MCF-10A cells [**Figure 2(III)** **, lane 1**]. Nestin and β3-tubulin mRNA transcripts were also detected in the two metastatic human glioblastoma cell lines U87 and D54 [**Figure 2(III)** **, lanes 7–8**]. The glial cell marker GFAP mRNA transcript was only detected in SKBR3 and MDA-MB-231 cells [**Figure 2(III)** **, lanes 3–4**]. While we failed to detect GFAP mRNA expression in cultured MDA-MB-435, U87 and D54 cells [**Figure 2(III)** **, lanes 5, 7–8**], we observed abundant specific GFAP protein expression in these cell lines by immunofluorescent (IFC) and immunohistochemistry (IHC) analyses (**Figure 3**).

These observations demonstrate that co-expression of multi-lineage differentiation markers, including epithelial, melanocytic and neuron/glial occurs in human breast cancer cell lines. Similar findings were observed in MDA-MB-435-GFP, MDA-MB-435-GFP-L cell lines and in MDA-MB-435-GFP cell lines derived from expansion of a single MDA-MB-435-GFP cell (**data not shown**). Thus, expression of melanocytic and neuronal/glial differentiation markers in our analysis could not be due to contamination of the MDA-MB-435 culture stock we used in this study with either melanoma or glioblastoma cancer cells.

To confirm our novel findings on neuronal/glial differentiation based on RT-PCR analyses, we conducted IFC and IHC analyses of the protein expression of lineage markers in cultured MDA-MB-435 cells (**Figure 3**). Consistent with the lack of cytokeratin mRNA expression in MDA-MB-435 cells [**Figure 2(I)** **, lane 5**], we failed to detect pan-cytokeratin protein expression in embedded MDA-MD-435 cells by IHC staining (**Figure 3B**). Compared to the Ul-MeL melanoma cell line that expressed high levels of melan-A (**Figure 3A**), the protein expression level of this melanocytic differentiation marker was considerably lower in MDA-MD-435 and U87 cells (**Figure 3A**). In contrast, cultured MDA-MB-435 cells strongly expressed both GFAP and nestin proteins. Similar patterns of IHC staining of GFAP and nestin were observed (**Figure 3**). The discrepancy between mRNA and protein expression of cytokeratin and GFAP could be due to differences in mRNA stability among genes analyzed. This observation highlights the importance of using multiple complementary analyses for drawing conclusions from gene expression assessment.

Further, we also observed aberrant transdifferentiation or lineage infidelity in the Ul-MeL melanoma cell line that co-expresses epithelial and neuronal markers [**Figure 2(I)** **(III), lanes 6**], and in two glioblastoma tumor cell lines that co-express epithelial and melanocytic markers [**Figure 2(I)** **(II), lanes 7–8**]. These findings indicate that terminal differentiation to the anticipated cellular type is altered in the cancerous state and that the phenomena of lineage infidelity that is associated with the ability of cancer cells to transdifferentiate, occurs in different cancer types and is not limited to breast cancer.

### Co-expression of multi-lineage protein markers in MDA-MB-435 primary tumors and metastases

**Figure 4:**
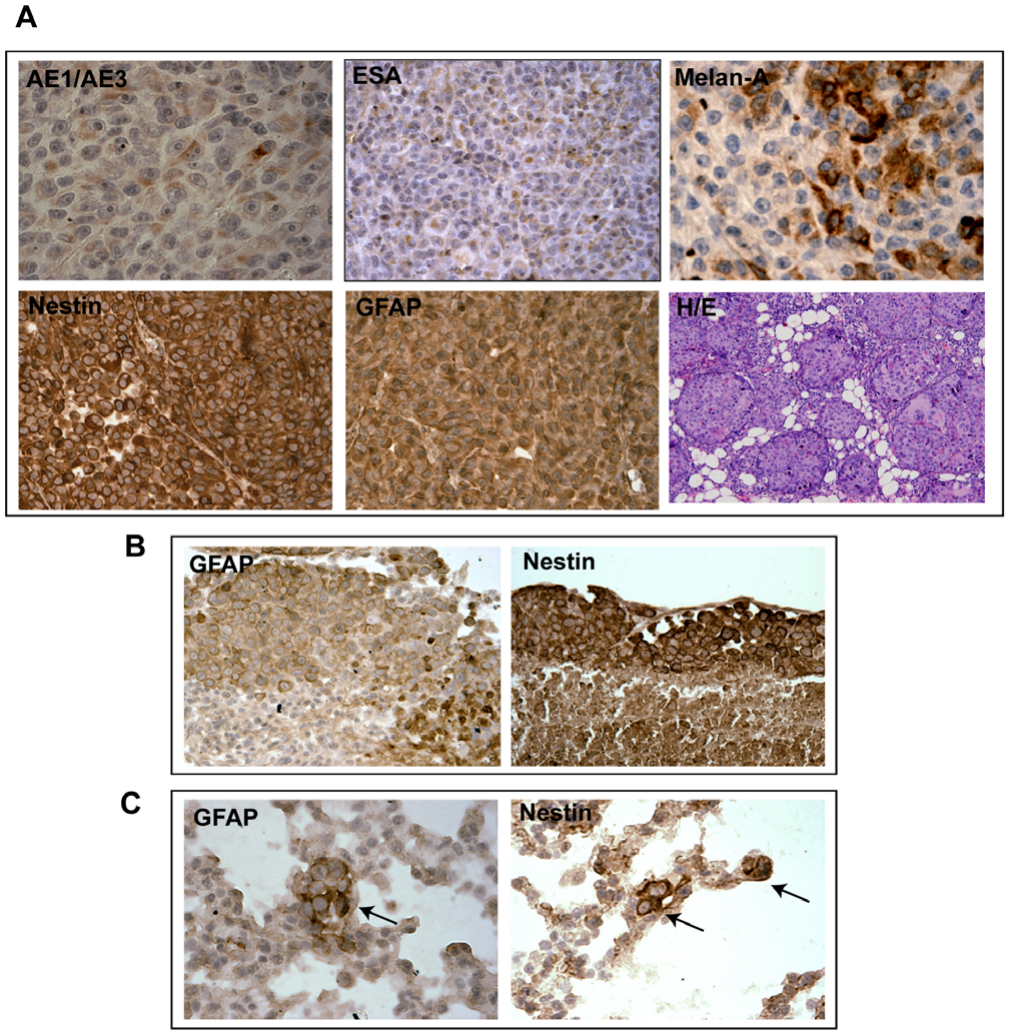
Determination of protein expression of lineage markers in MDA-MB-435 xenografts and in metastatic lesions. Immunohistochemical detection of epithelial (AE1/AE3, ESA), melanocytic (melan-A), and neuronal/glial markers (GFAP, nestin) in orthotopic mammary fat pad tumors, and mammary duct differentiation (H&E) (**A**), pleural lung macrometastases (**B**) and lung micrometastases (**C**, arrows).

To further confirm that the expression of epithelial, melanocytic and neuronal/glial markers in MDA-MB-435 cells is not due to an artifact of cell culture, MDA-MB-435 primary tumors growing orthotopically in the mammary fat pad (m.f.p) of nude mice and pleural macro- and micro-metastases of MDA-MB-435 were utilized for the analyses of the expression of three cellular lineage markers by IHC (**Figure 4** and **Methods**). We consistently observed more robust and consistent MDA-MB-435 tumor growth (higher tumor cell take rate, initiation of tumor growth, higher level of tumor angiogenesis and higher incidence of lung metastasis) at the m.f.p, than when MDA-MD-435 was transplanted under the skin of the hind leg or on the back. These observations demonstrate that the m.f.p represents a more favorable microenvironment for MDA-MB-435 tumor initiation, expansion and progression, consistent with its ability to undergo mammary epithelial differentiation [9], [14]. While cultured MDA-MB-435 cells were negative for AE1/AE3 staining for pan-cytokeratin [**Figure 2(I)** **, lane 5**], MDA-MB-435 primary tumors exhibited weakly positive staining for AE1/AE3 (**Figure 4A**) and strong staining for ESA. This observation is consistent with a previous report [11]. Additionally, we also observed evidence of mammary duct differentiation at the periphery of MDA-MB-435 tumors (**Figure 4A**, H&E panel). Similar to IFC, IHC and PCR analyses *in vitro*, both the orthotopic primary tumor and macro/micro lung metastases displayed intense and homogenous expression of nestin and GFAP (**Figure 4**). In contrast, strong melan-A staining was only detected in discrete clusters of cancer cells within the primary tumor (**Figure 4A**).

Based on the close clustering of MDA-MB-435 cells with melanoma cell lines in microarray analysis [5], the possibility of the breast cancer patient, from whom MDA-MB-435 was derived from, having an undetected occult melanoma was suggested. However, cell lines generated from breast tumors and from non-small lung carcinoma used in the same micro-array study also failed to yield a clear clustering pattern according to their tissue of origin. In particular, two other invasive breast cancer cell lines, Hs578T and BT-549 were clustered together with brain tumor cell lines, a finding that is consistent with our detection of neuronal and glial differentiation markers in breast cancer cell lines. Further, a recent report demonstrated a wide spectrum of expression of melanocyte-related genes in histologically confirmed human breast tumor specimens [3]. It thus appears that aberrant co-expression of multi-lineage markers via transdifferentiation or lineage infidelity occurs frequently in breast cancer. Therefore, molecular signatures derived from gene expression profiling should not be used as exclusive evidence or criteria to determine the tissue origin of a cancer cell line or a metastatic lesion from an occult primary tumor. Functional properties such as *in vitro* functional characterization of cellular differentiation (acinus formation in 3D gel culture, production of milk products upon induction of lineage-specific differentiation), *in vivo* tumor growth and progression should all be taken into account when we consider the classification of a human cancer cell line.

We have demonstrated in this report the co-expression of three ectoderm cell lineage differentiation markers by a panel of breast cancer cell lines, by MDA-MB-435 cells obtained from the ATCC, by cell lines derived from single-cell cloning of MDA-MB-435 and MDA-MB-435 cells derived from lung metastases grown *in vitro*; and as well as by MDA-MB-435 orthotopic primary tumors and lung metastases *in vivo*. It is thus highly unlikely that this cell line was contaminated by both melanoma cells and neuron/glial cancer cells, or that the breast cancer patient from whom MDA-MB-435 cells were derived also had an undiagnosed melanoma and undiagnosed glioblastoma.

In conclusion, our observations indicate that aberrant multi-lineage transdifferentiation or lineage infidelity occurs frequently in multiple types of human cancer and may be a wide spread phenomenon.
